# Quantitative Profiling of Arabidopsis Polar Glycerolipids under Two Types of Heat Stress

**DOI:** 10.3390/plants9060693

**Published:** 2020-05-29

**Authors:** Feng Qin, Liang Lin, Yanxia Jia, Weiqi Li, Buzhu Yu

**Affiliations:** 1 The Germplasm Bank of Wild Species, Kunming Institute of Botany, Chinese Academy of Sciences, Kunming 650201, China; qinfeng@mail.kib.ac.cn (F.Q.); linliang@mail.kib.ac.cn (L.L.); jiayanxia@mail.kib.ac.cn (Y.J.); 2 University of the Chinese Academy of Sciences, Beijing 100049, China

**Keywords:** lipidomics, glycerolipids, heat shock, moderate heat stress, HSP101, prokaryotic pathway, eukaryotic pathway

## Abstract

At the cellular level, the remodelling of membrane lipids and production of heat shock proteins are the two main strategies whereby plants survive heat stress. Although many studies related to glycerolipids and HSPs under heat stress have been reported separately, detailed alterations of glycerolipids and the role of HSPs in the alterations of glycerolipids still need to be revealed. In this study, we profiled the glycerolipids of wild-type Arabidopsis and its HSP101-deficient mutant *hot-1* under two types of heat stress. Our results demonstrated that the alterations of glycerolipids were very similar in wild-type Arabidopsis and *hot-1* during heat stress. Although heat acclimation led to a slight decrease of glycerolipids, the decrease of glycerolipids in plants without heat acclimation is more severe under heat shock. The contents of 36:x monogalactosyl diacylglycerol (MGDG) were slightly increased, whereas that of 34:6 MGDG and 34:4 phosphatidylglycerol (PG) were severely decreased during moderate heat stress. Our findings suggested that heat acclimation could reduce the degradation of glycerolipids under heat shock. Synthesis of glycerolipids through the prokaryotic pathway was severely suppressed, whereas that through the eukaryotic pathway was slightly enhanced during moderate heat stress. In addition, HSP101 has a minor effect on the alterations of glycerolipids under heat stress.

## 1. Introduction

Heat stress, which is one of the most common abiotic stresses experienced by plants, can affect the distribution of wild plants and the production of crops [1,2,3,4,5]. According to the extent and rate of the change in temperature, heat stress can be classified into heat shock and moderate heat stress [6,7]. With the increasing output of greenhouse gases, particularly CO_2_, the temperature has increased by approximately 0.6 °C during the past 100 years and is projected to continue to rise at a high rate [8]. According to previous research, a temperature increase of 3–4 °C could cause crop yields to fall by 15%–35% in Africa and Asia and by 25%–35% in the middle east [9]. Field experiments have indicated that an increased temperature will have a net negative impact on the yield of rice in tropical/subtropical Asia [10]. An increase in temperature of only 1 °C in the wheat-growing season reduces wheat yields by 3%–10% in China [11]. Due to the escalating adverse impact of high temperature on agricultural crop production, we should investigate all aspects of the underlying mechanisms related to heat stress.

Plant cellular membranes have long been proposed to be one of the prime targets of temperature stress [6,12,13,14,15,16,17]. It is generally accepted that maintaining the fluidity and integrity of membranes is of fundamental importance for plants to survive stress [18]. Glycerolipids are the major constituents of membranes, and they can be divided into two categories, plastidic lipids and extraplastidic lipids, based on their distribution [15,19]. Plastidic lipids, including monogalactosyl diacylglycerol (MGDG), digalactosyl diacylglycerol (DGDG), and phosphatidylglycerol (PG), are the main constituents of chloroplast membranes, and extraplastidic lipids, including phosphatidylcholine (PC), phosphatidylethanolamine (PE), phosphatidylinositol (PI), phosphatidylserine (PS), and phosphatidic acid (PA), are the main constituents of plasma membranes [19,20]. To maintain membrane functions, changes in the composition and unsaturation of glycerolipids occur under temperature stresses [13,17,19,20]. Low temperature leads to a decrease in membrane fluidity; however, owing to the increases in many glycerolipid species with polyunsaturated acyl chains, such as 36:6 PC, 36:6 PE, 34:6 MGDG, and 36:6 DGDG, the fluidity of the membrane is maintained in Arabidopsis during cold acclimation [17,19]. The conversion of MGDG to DGDG and oligogalactolipids under freezing stress, which is catalyzed by SENSITIVE TO FREEZING 2 (SFR2), not only prevents the formation of nonbilayer-type structures but also increases the repulsive hydration force between apposed bilayers during freeze-induced dehydration [21,22,23]. The response of glycerolipids to moderate heat stress has also been intensively investigated [6,13,14,16,24,25]. Plants show an increase in 18:2 (linoleate)-containing galactolipids and a decrease in 18:3 (α-linoleate)-containing galactolipids in chloroplasts; increases in phospholipids containing 16:0 (palmitate), 18:0 (stearate), and 18:1 (oleate) in the endoplasmic reticulum and plasma membrane; and an increase in triacylglycerol containing 18:3 and 16:3 (hexadecatrienoic acid) as lipid droplets under moderate heat stress [6,13,14,24]. Recent studies have highlighted the importance of MGDG catabolism under moderate heat stress [12]. Although many lipidomic studies have been reported, more details still need to be elucidated during moderate heat stress. In addition, the response of glycerolipids to heat shock has never been reported.

In addition to the changes in membrane lipids, the production of HSPs plays an important role during heat stress [26,27,28,29,30]. Previous research indicated that changes in membrane fluidity could induce the production of HSPs [18]. HSPs mainly function as molecular chaperones to prevent the aggregation of denatured proteins, assist in the folding of nascent polypeptides, aid in the refolding of denatured proteins, or help to resolubilize aggregated denatured proteins [26,31]. Therefore, the *hot-1* Arabidopsis mutant, which has a mutation in the HSP101 gene, is more sensitive to high-temperature stress than the wild-type Arabidopsis [32,33]. Previous studies have indicated that some small HSPs also function as membrane stabilizers under high-temperature stress [27,34]. Hsp17 can antagonize the heat-induced hyperfluidization of the membrane and thereby help to preserve the structural and functional integrity of biomembranes in *Synechocystis* [27]. However, studies concerning the effect of HSPs on the content and alteration of glycerolipids under two different types of heat stress have never been reported.

In this work, we examined the leaf glycerolipids of wild-type Arabidopsis (Col) and its HSP101 T-DNA insertion mutant *hot-1* under two types of heat stress: Moderate heat stress (MHS; 30 °C, 18 days) and heat shock (HS; 45 °C, 3 h), which have commonly been applied in previous studies [6]. A lipidomic analysis based on electrospray tandem mass spectrometry (ESI-MS/MS) was used to profile the molecular species of glycerolipids [17]. Although heat acclimation, a process including the pre-exposure of plants to sublethal temperatures, led to a slight decrease in glycerolipids, it reduced the degradation of glycerolipids under heat shock. However, the remodelling of plastidic lipids, severely suppressed the prokaryotic pathway and slightly enhanced the eukaryotic pathway, that occurred during moderate heat stress. Our research also indicated that HSP101 had a minor effect on the alteration of membrane lipids under both types of heat stress.

## 2. Results

### 2.1. Contents and Alterations of Glycerolipids During the Heat Acclimation Process in Wild-Type Arabidopsis and Hot-1

**Figure 1:**
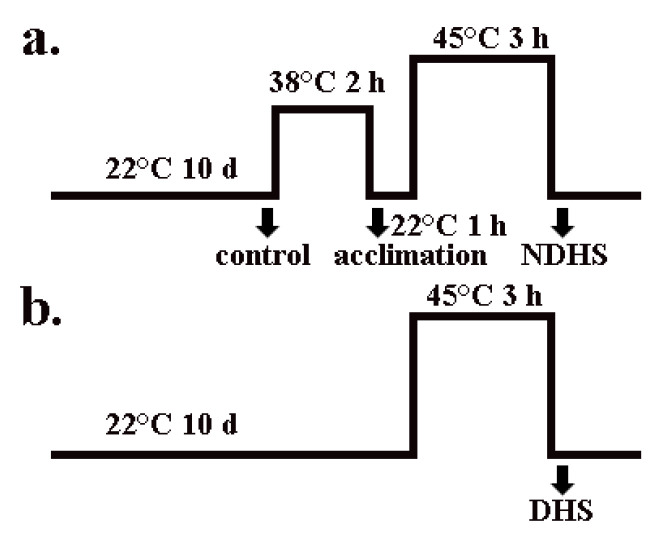
Diagrammatic sketch of the heat shock process. (**a**) The process of heat shock with heat acclimation; (**b**) the process of heat shock without heat acclimation. The black arrow indicates where the samples were harvested. NDHS: Non-direct heat shock; DHS: Direct heat shock.

**Figure 2:**
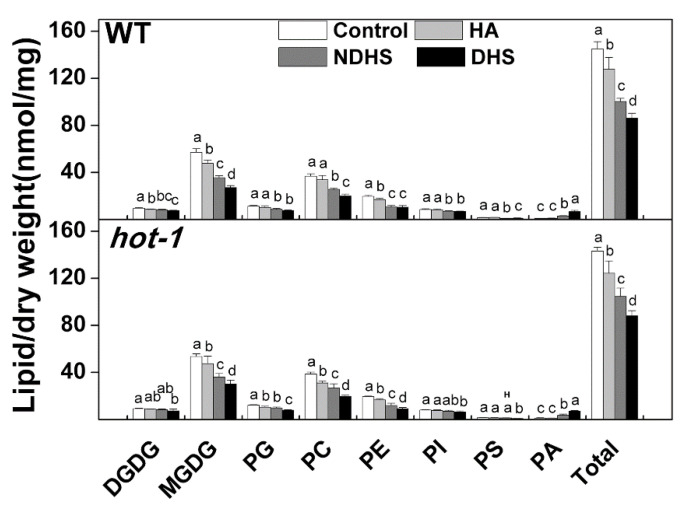
Effects of heat shock on the total content of glycerolipids in various head group classes in wild-type Arabidopsis and *hot-1*. Values shown are the mean ± SE; *n* = 4 or 5. Means with different letters are significantly different according to the least significant difference (LSD) test at *P* < 0.05 under heat shock. The letter “H” indicates that the amount of lipid was significantly higher (*P* < 0.05) in *hot-1*, compared to wild-type Arabidopsis at the corresponding treatment. HA: Heat acclimation; NDHS: Heat shock with heat acclimation; DHS: Direct heat shock.

Previous studies have mainly attributed the increased thermotolerance of plants to the accumulation of HSPs during heat acclimation. However, whether and how membrane lipids respond to this process has seldom been investigated [32,33,35]. In addition, whether HSPs affect membrane lipids during this process is also not clear. In the heat shock experiments, neither wild-type Arabidopsis nor *hot-1* could survive without heat acclimation, whereas wild-type Arabidopsis survived with heat acclimation (Figure S1) [36]. We profiled glycerolipids throughout the whole process: Control, heat acclimation, non-direct heat shock (NDHS, heat shock after heat acclimation), and direct heat shock (DHS, heat shock without acclimation) (Figure 1). A glycerolipids analysis indicated that the contents and alterations of glycerolipids were very similar in wild-type Arabidopsis and *hot-1* after heat acclimation (Figure 2). In wild-type Arabidopsis and *hot-1*, the content of total glycerolipids decreased by approximately 11.87% and 13.15% separately after heat acclimation (Figure 2). In wild-type Arabidopsis, the contents of PG, PC, PS, PI, and PA did not change after heat acclimation, whereas that of MGDG, PE, and DGDG decreased. The contents of MGDG, PE, and DGDG decreased by approximately 16.49%, 14.60%, and 10.24% separately. In *hot-1*, the contents of PI, PS, PA, and DGDG did not change after heat acclimation, but that of MGDG, PG, PC, and PE decreased. The contents of MGDG, PG, PC, and PE decreased by approximately 11.65%, 15.07%, 19.83%, and 14.20% separately. Overall, heat acclimation led to a decrease in glycerolipids in both wild-type Arabidopsis and *hot-1*.

### 2.2. Heat Acclimation Reduced the Degradation of Glycerolipids During Heat Shock in Wild-Type Arabidopsis and Hot-1

After heat shock, the content of total glycerolipids decreased dramatically in both wild-type Arabidopsis and *hot-1*, regardless of whether the plants were acclimated or not, except that the content of PS was slightly higher after NDHS in *hot-1* (Figure 2). This decrease involved almost all classes of glycerolipids, except for PA. However, compared to NDHS, DHS led to more severe degradation: The total lipid content decreased 40.50% after DHS and 30.83% after NDHS in wild-type Arabidopsis; the total lipid content decreased 38.42% after DHS and 26.97% after NDHS in *hot-1* (Figure 2). In wild-type Arabidopsis, the more severe degradation observed after DHS involved MGDG and PC. The content of MGDG and PG decreased by approximately 37.71% and 30.28% separately after NDHS, but decreased by approximately 52.54% and 45.70% separately after DHS. In *hot-1*, the more severe degradation observed after DHS involved MGDG, PG, PC, and PE. The content of MGDG, PG, PC, and PE decreased by approximately 32.68%, 19.91%, 31.05%, and 39.09% separately after NDHS, but decreased by approximately 43.80%, 35.50%, 49.46%, and 52.71% separately after DHS. In contrast, the contents of PA after DHS were greater than those after NDHS in both wild-type Arabidopsis and *hot-1* (Figure 2). The content of PA increased by approximately 258.39% and 236.18% separately after NDHS in wild-type Arabidopsis and *hot-1*, but increased approximately 684.11% and 570.84% separately after DHS. Many molecular species of PA that did not exist in the control or after heat acclimation appeared after heat shock, such as 34:4, 34:5, and 34:6 PA (Figure S2). All molecular species in each class of glycerolipids showed similar changes during heat shock in both wild-type Arabidopsis and *hot-1* (Figures S2 and S3). Overall, the degradation of glycerolipids in plants without heat acclimation is more severe under heat shock.

### 2.3. The Contents of Glycerolipids Changed Profoundly Under Moderate Heat Stress in Wild-Type Arabidopsis and Hot-1

**Figure 3:**
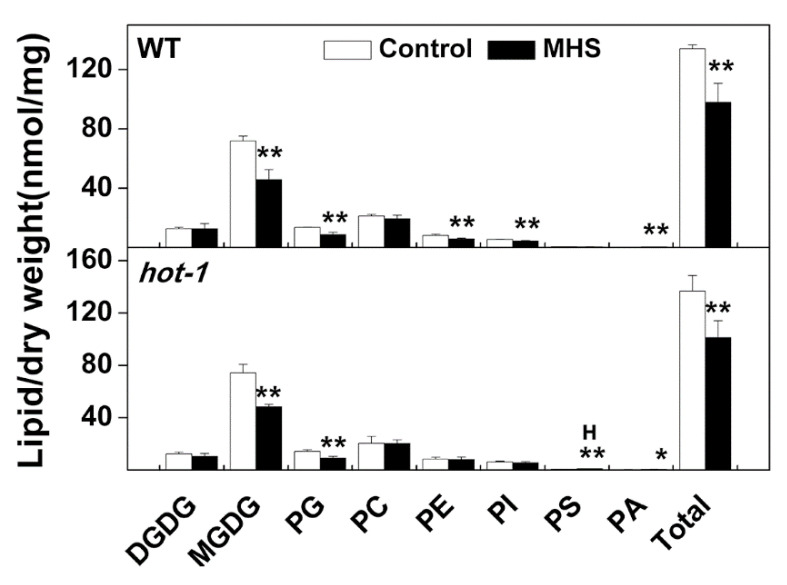
Effects of moderate heat stress on the total content of glycerolipids of various head group classes in wild-type Arabidopsis and *hot-1*. Values shown are the mean ± SE; *n* = 4 or 5. Symbols * and ** indicate that the content of glycerolipids under moderate heat stress differed significantly from that under ambient temperature in wild-type Arabidopsis and *hot-1* at *P* ≤ 0.05 and ≤ 0.01, respectively. The letter “H” indicates that the amount of lipid was significantly higher (*P* < 0.05) in *hot-1*, compared to wild-type Arabidopsis at the corresponding treatment. MHS: Moderate heat stress.

Although there is no significant difference in phenotypes between wild-type Arabidopsis and *hot-1* under ambient temperature, it seems that wild-types of Arabidopsis grew better than *hot-1* under moderate heat stress (Figure S4). To investigate whether moderate heat stress and HSP101 affect the alteration of glycerolipids, we profiled glycerolipids in the leaves of wild-type Arabidopsis and *hot-1* under ambient temperature and moderate heat stress (Figure 3). We found that the content of glycerolipids changed profoundly in wild-type Arabidopsis and *hot-1* after moderate heat stress. However, the contents of glycerolipids were very similar in wild-type Arabidopsis and *hot-1*, except that of PS are slightly higher in *hot-1* after moderate high temperature (Figure 3). In wild-type Arabidopsis, the content of total glycerolipids decreased by approximately 26.91% after moderate heat stress. This decrease in total glycerolipids mainly involved plastidic lipids, especially MGDG and PG. The contents of MGDG and PG decreased by approximately 36.20% and 35.24%, respectively. While the content of extraplastidic lipids decreased by less than that of plastidic lipids, the content of PE and PI decreased by approximately 28.76% and 19.27%, respectively. In *hot-1*, the content of total glycerolipids decreased by approximately 26.02% after moderate heat stress. This decrease is mainly due to the decrease of MGDG and PG, which decreased by approximately 34.62% and 36.43% separately. However, the content of PE and PI did not change after moderate heat stress. In contrast, the content of PA increased slightly after moderate heat stress in both wild-type Arabidopsis and *hot-1*. Overall, the moderate heat stress led to a decrease in total glycerolipids, which was mainly due to the decrease in plastidic lipids, such as MGDG and PG.

### 2.4. The Alterations of Plastidic Lipids Molecular Species under Moderate Heat Stress in Wild-Type Arabidopsis and Hot-1

**Figure 4:**
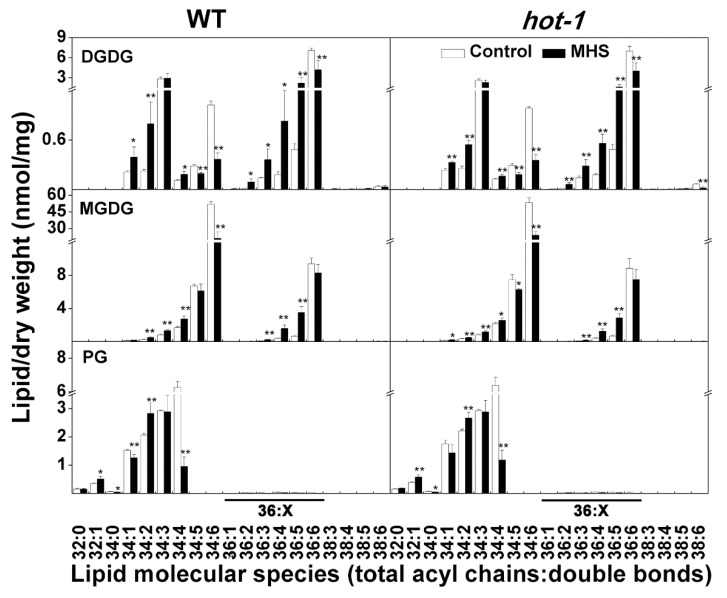
Effects of moderate heat stress on plastidic lipid molecular species in wild-type Arabidopsis and *hot-1*. Values shown are the mean ± SE; *n* = 4 or 5. Symbols * and ** indicate that the content of glycerolipids under moderate heat stress differed significantly from that under ambient temperature in wild-type Arabidopsis and *hot-1* at *P* ≤ 0.05 and ≤ 0.01, respectively. MHS: Moderate heat stress.

Previous studies have indicated that plastidic lipids are more sensitive to environmental stress than extraplastidic lipids [15]. However, the response of individual plastidic lipid molecular species to moderate heat stress is not clear. Our data indicated that the contents and alteration of individual plastidic lipid molecular species were almost identical in wild-type Arabidopsis and *hot-1* after moderate heat stress (Figure 4). Although the content of MGDG decreased, the content of 36:x MGDG, which contains 36 carbons in two acyl chains and is mainly synthesized through the eukaryotic pathway, increased by approximately 29.71% and 18.16% separately under moderate heat stress in wild-type Arabidopsis and *hot-1* (Figure 4). This increase involved all 36:x MGDG (total acyl chains: Double bonds) molecular species except for 36:6 MGDG. The content of 34:6 MGDG, which is synthesized through the prokaryotic pathway, decreased by approximately 58.81% and 54.67% separately under moderate heat stress in wild-type Arabidopsis and *hot-1*. However, the other 34:x MGDG molecular species showed slight increases or no change during moderate heat stress. In contrast to the change in MGDG, the total DGDG content did not change under moderate heat stress in both wild-type Arabidopsis and *hot-1* (Figure 3). However, the DGDG composition underwent a profound alteration (Figure 4). The contents of 36:6 and 34:6 DGDG, both of which have more double bonds, decreased significantly in both wild-type Arabidopsis and *hot-1*. Other molecular species, such as 36:5, 36:4, and 34:2 DGDG, all increased during moderate heat stress. The decrease in total PG was mainly due to the decrease in 34:4 PG, which is also synthesized through the prokaryotic pathway, in both wild-type Arabidopsis and *hot-1*. From the observed alteration of plastidic lipids, we know that the molecular species of plastidic lipids changed profoundly during moderate heat stress.

### 2.5. The Alterations of Extraplastidic Lipids Molecular Species Under Moderate Heat Stress in Wild-Type Arabidopsis and Hot-1

**Figure 5:**
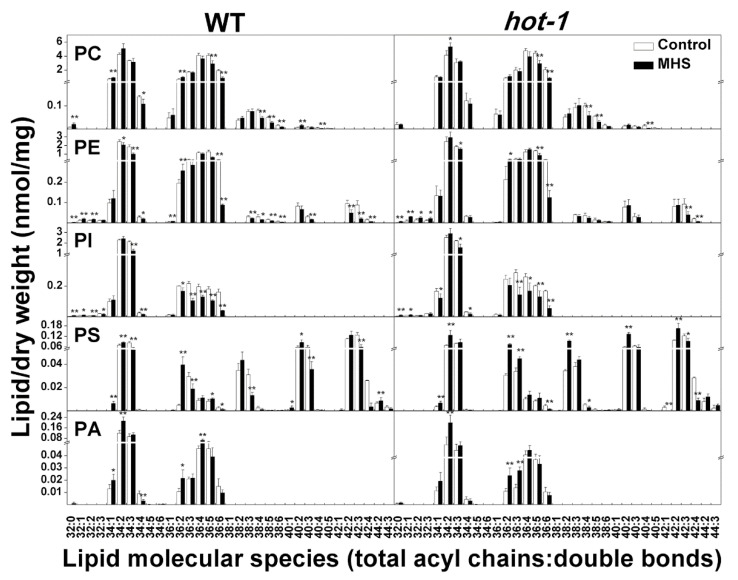
Effects of moderate heat stress on extraplastidic lipid molecular species in wild-type Arabidopsis and *hot-1*. Values shown are the mean ± SE; *n* = 4 or 5. Symbols * and ** indicate that the content of glycerolipids under moderate heat stress differed significantly from that under ambient temperature in wild-type Arabidopsis and *hot-1* at *P* ≤ 0.05 and ≤ 0.01, respectively.

Under moderate heat stress, the changes in extraplastidic lipids were less pronounced than those in plastidic lipids, but some interesting alterations still occurred (Figure 5). First, the molecular species containing 34:3 (including 18:3 and 16:0) fatty acids all showed decreases in PE, PI, and PS, by approximately 45.09%, 40.05%, and 37.2%, respectively in wild-type Arabidopsis. In contrast, the molecular species containing 34:3 fatty acids only decreased in PE and PI, by approximately 15.22% and 28.30%, respectively in *hot-1*. Second, the molecular species containing 36:5 and 36:6 fatty acids (including 18:2/18:3 and 18:3/18:3) decreased in almost all classes of extraplastidic lipids, with the exception of PA and PS, in both wild-type Arabidopsis and *hot-1*. Third, moderate heat stress did not lead to a dramatic change in PA in wild-type Arabidopsis and *hot-1*. The increase in PA was mainly due to the production of 34:2 PA, whereas 34:3, 36:5, and 36:6 PA showed no change under moderate heat stress. Last, the molecular species of PS, including 34:2 PS, 36:2 PS, 38:2 PS, 40:2 PS, and 42:2 PS, increased significantly in *hot-1* under moderate heat stress. Whereas, that in wild-type Arabidopsis increased less or did not change, except for 36:2 PS. Overall, moderate heat stress seemed to mainly lead to a decrease in more unsaturated extraplastidic lipids containing 18:3 acyl chains in wild-type Arabidopsis and *hot-1*. 

### 2.6. Changes in the Double Bond Index Under the Two Types of Heat Stress in Wild-Type Arabidopsis and Hot-1

**Figure 6:**
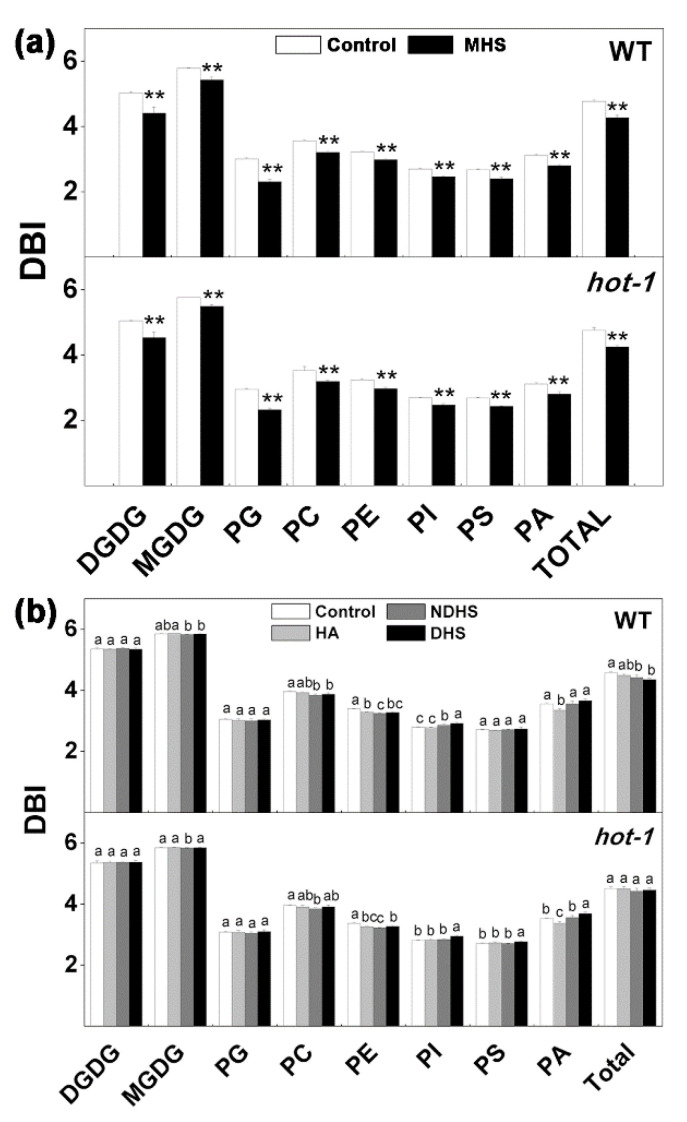
Effects of two types of heat stress on the DBI of glycerolipids in wild-type Arabidopsis and *hot-1*. (**a**) The alteration of DBI in wild-type Arabidopsis and *hot-1* under moderate heat stress. (**b**) The alteration of DBI in wild-type and *hot-1* under heat shock. DBI = (Σ[N × nmol/mg])/Σ nmol/mg, where *N* is the total number of double bonds in the two fatty acid chains of each glycerolipid molecule. Values shown are the mean ± SE; *n* = 4 or 5. Symbols * and ** indicate that the content of glycerolipids under moderate heat stress differed significantly from that under ambient temperature in wild-type Arabidopsis and *hot-1* at *P* ≤ 0.05 and ≤ 0.01, respectively. Means with different letters are significantly different according to the least significant difference (LSD) test at *P* < 0.05 under heat shock.

Previous studies have indicated that temperature can induce a change in the double bond index (DBI), with high temperature leading to a decrease in the DBI and low temperature leading to an increase [24,37,38,39]. Our results were consistent with previous studies: The DBI of total lipids decreased by approximately 10.47% and 10.72% separately under moderate heat stress in wild-type Arabidopsis and *hot-1* (Figure 6a). A careful analysis indicated that the DBI of each class of glycerolipids all decreased under moderate high temperature in both wild-type Arabidopsis and *hot-1* (Figure 6a). Among plastidic lipids, the DBI of PG decreased the most in both wild-type Arabidopsis and *hot-1*, by about 23.00% and 21.29%, respectively. In contrast, the DBI of MGDG decreased the least in both wild-type Arabidopsis and *hot-1*, about 6.07% and 4.68%, respectively. The DBI of all extraplastidic lipids decreased by approximately the same extent in both wild-type Arabidopsis and *hot-1* under moderate heat stress (Figure 6a). Under heat shock, the DBI of glycerolipids changed slightly in both wild-type Arabidopsis and *hot-1* (Figure 6b). The DBI of total glycerolipids did not change during heat acclimation and decreased slightly after NDHS or DHS in wild-type Arabidopsis, whereas the DBI of total glycerolipids did not change during the whole process of heat shock. 

### 2.7. The Alterations of LysoPLs under Two Types of Heat Stress in Wild-Type Arabidopsis and Hot-1

**Figure 7:**
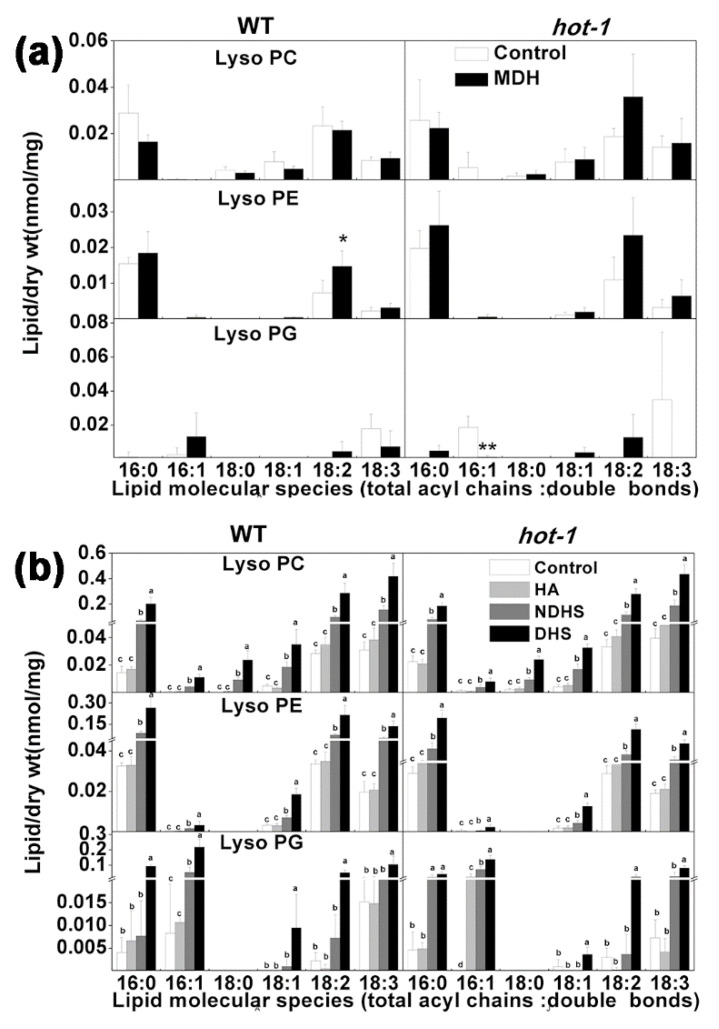
Effects of two types of heat stress on the content of lysolipids in wild-type Arabidopsis and *hot-1*. (**a**) The alteration of lysolipids under moderate heat stress. (**b**) The alteration of lysolipids under heat shock. Values shown are the mean ± SE; *n* = 4 or 5. Symbols * and ** indicate that the content of LysoPLs under moderate heat stress differed significantly from that under ambient temperature in wild-type Arabidopsis and *hot-1* at *P* ≤ 0.05 and ≤ 0.01, respectively. Means with different letters are significantly different according to the least significant difference (LSD) test at *P* < 0.05 under heat shock.

Previous studies have confirmed that LysoPLs act as secondary signal messengers in plant signalling [40,41,42]. They are involved in many important physiological processes, such as cell elongation, shoot gravitropism, and pollen maturation [43]. The changes in lysophospholipids under heat stress were investigated in the present study (Figure 7). Under moderate heat stress, the content of all LysoPL species almost did not change in both wild-type Arabidopsis and *hot-1*, except 18:2 Lyso PE in wild-type Arabidopsis and 16:1 LysoPG in *hot-1* (Figure 7a). Under heat acclimation, the content of LysoPLs also did not increase. However, the content of LysoPL species increased dramatically after heat shock (both NDHS and DHS) in both wild-type Arabidopsis and *hot-1* (Figure 7b). The increase in the content of LysoPLs after NDHS was lower than the increase in LysoPLs after DHS. The increase in the content of LysoPLs involved all LysoPLs species (Figure 7b). These results indicated that LysoPLs respond actively to heat shock.

## 3. Discussion

Although many studies related to how glycerolipids respond to high-temperature stress have been reported, a number of questions still remain to be addressed [13,14,16,24,25]. In this study, we quantitatively profiled the molecular species of glycerolipids in the leaves of wild-type Arabidopsis and *hot-1* plants under two types of heat stress, heat shock (HS; 45 °C, 3 h), and moderate heat stress (MHS; 30 °C, 18 days). The glycerolipid data indicated that HSP101 has a minor effect on the alterations of glycerolipids under both types of heat stress. Although heat acclimation led to a decrease in some classes of glycerolipids, it reduced the degradation of glycerolipids under heat shock. Under moderate heat stress, the alterations of the contents of individual molecular species of glycerolipids demonstrated that the prokaryotic and eukaryotic pathways are closely coordinated to meet the overall demands for homeostasis maintenance.

Previous studies have demonstrated that HSP101 plays an important role in acquired thermotolerance, and whether HSP101 has an effect on the metabolism of glycerolipids under heat stress has not previously been investigated [32,33]. Judging from the glycerolipids data obtained in our study, the contents and alterations of glycerolipids were very similar between wild-type Arabidopsis and *hot-1* under the two types of heat stress. The only difference was that in comparison to wild-type Arabidopsis, *hot-1* exhibited a higher content of PS after NDHS and moderate heat stress (Figure 2, Figure 3). PS is a kind of membrane glycerolipid that contains very long chain fatty acids [44,45]. Its outward movement could disrupt the asymmetry of the membrane and trigger cell death [46]. Our previous research also suggested that the length of its acyl chains may be correlated with the plant lifespan [45]. The higher content of PS in *hot-1* implies that *hot-1* may suffer more negative impacts than wild-type Arabidopsis under NDHS and moderate heat stress. Muller et al. also has revealed that heat shock transcription factors (HSF), which could mediate amplification of heat-responsive genes, such as HSPs, did not affect the accumulation of triacylglycerols during heat acclimation [6]. It seems that the metabolism of glycerolipids was not dependent on HSFs during heat stress. Although HSP101 plays a minor role in the alteration of glycerolipids during heat stress, we assume that it may function in the repair of membrane lipids after heat stress, since HSP101 can protect proteins, including those involved in the synthesis of membrane lipids, from denaturation during heat shock [31].

The acclimation process can increase the tolerance of plants to extreme temperatures [17,19,32]. Previous studies have demonstrated that the remodelling of glycerolipids, including increases in their unsaturation and contents, occurs during cold acclimation and contributes to freezing tolerance [17,19]. Our results demonstrated that heat acclimation (38 °C, 2 h) led to decreased contents of MGDG and PG, particularly MGDG, in both wild-type Arabidopsis and *hot-1*. MGDG is the most sensitive glycerolipid during environmental stresses such as drought, freezing, and dehydration [12,23,47]. Since MGDG tends to form a hexagonal II phase or other nonbilayer phases, a decrease in MGDG could maintain the integrity of the membrane during heat shock [48]. The decreased MGDG may be converted into triacylglycerols, which accumulated during heat acclimation in the previous report [35]. The contents of PA and LysoPLs did not increase during heat acclimation. PA and LysoPLs have been recognized as secondary signal messengers in plant signalling, and their contents increase dramatically under environmental stress [40,41]. The DBI of glycerolipids also did not change during heat acclimation. These clues suggest that the alteration of glycerolipids during heat acclimation may only have a minor effect on acquired thermotolerance in our study. The reason for this small effect may have been that the time for heat acclimation was too short (only 2 h) to alter the glycerolipids to an extent that could lead to acquired thermotolerance. Our results are consistent with previous research showing that the time required for the fatty acid composition to adjust to high-temperature growth conditions is more than 60 h, which corresponds to the occurrence of new lipid synthesis and turnover [24]. Although heat acclimation has little effect on the alteration of glycerolipids, it reduces the degradation of glycerolipids under heat shock. According to the above discussion, neither the alteration of glycerolipids during heat acclimation nor HSP101 led to reduced degradation after heat shock. The observed decrease in degradation may have been due to the production of some HSPs during acclimation, since some studies have proven that HSPs can be inserted into the cell membrane to maintain the integrity of membranes [34,49]. However, this assumption needs to be further verified, since the effect of HSPs on the plant membrane has never been reported.

The alterations of glycerolipids in Arabidopsis leaves under moderate heat stress have been reported in several studies [6,13,14,25]. However, these studies only showed the alteration of the relative composition of glycerolipids, whereas the changes in the absolute contents of glycerolipids have seldom been reported in detail [13,14,24]. Indeed, data including the changes in the composition of glycerolipids could reflect rules of the alteration of glycerolipids under moderate heat stress [13]. However, data on the changes in the absolute contents of glycerolipids could provide more information. Our data demonstrated that the content of glycerolipids decreased during moderate heat stress. This result suggested that the synthesis of glycerolipids was hampered during moderate heat stress. The decrease was mainly due to the decrease in plastidic lipids. It seems that the synthesis of extraplastidic lipids, which occurs in the ER, is less affected [50,51]. A careful analysis indicated that the contents of 36:x MGDG, synthesized via the eukaryotic pathway, were increased, although this increase did not include 36:6 MGDG. The content of 36:x DGDG did not change during moderate heat stress. These results suggested that the synthesis of glycerolipids by the eukaryotic pathway in the ER was slightly increased. The dramatic decreases of 34:6 MGDG and 34:4 PG indicated that the prokaryotic pathway was severely hampered under moderate heat stress. In addition, the decrease in 18:3-containing molecular species, such as 36:6 DGDG, 34:6 DGDG, and 34:6 MGDG, and the increase in 18:2-containing molecular species, such as 36:5 DGDG, 34:5 DGDG, and 36:5 MGDG, implied that the desaturase that converts 18:2 to 18:3 fatty acids may be affected during moderate heat stress. In addition, some 18:3-containing molecular species may be hydrolyzed by PHOSPHOLIPASE1 (PLA1) to generate several jasmonates including OPDA, MeJA, JA, and JA-Ile, which contribute to many abiotic stresses tolerance, including thermotolerance [52,53,54,55]. Thus, moderate heat stress hampered the synthesis of glycerolipids mainly through a decrease in the prokaryotic pathway, whereas synthesis via the eukaryotic pathway was slightly increased. 

## 4. Materials and Methods

### 4.1. Plant Materials

*Arabidopsis thaliana* plants of the Columbia (Col) accession and its HSP101 T-DNA insertion mutant (salk-066374) *hot-1* were used in this study. The *hot-1* seeds were kindly provided by Prof. Jian Liu (Shandong Normal University, China), and the total absence of HSP101 was confirmed by a western blot assay [36].

### 4.2. Growth Conditions and Treatments

For the moderate heat stress experiment, we germinated seeds in soil. After two days at 4 °C, the seeds were germinated and grown at 22 °C for two weeks in a growth chamber. Then, a subset of these seedlings was transferred to a 30 °C growth chamber. Except for their different temperatures, the conditions of these two chambers were identical: 80% relative humidity, 120 μmol m^−2^ s^−1^ light, and a 12-h photoperiod. After 18 days, the leaves of these seedlings were harvested for lipid analysis and other experiments. For the heat shock experiments using seedlings in agar plates, we germinated seeds in 0.8% solidified agar plates containing a 1× Murashige and Skoog basal medium and 1% sucrose. After two days at 4 °C, the seeds were germinated and grown at 22 °C under 120 μmol m^−2^ s^−1^ light and a 12-h photoperiod for 10 days in a growth chamber. After 10 days, a subset of plants were transferred to 38 °C for 2 h for heat acclimation, followed by 1 h at 22 °C and then 3 h at 45 °C (NDHS), while the other subset of the seedlings were directly transferred to 45 °C for 3 h (DHS) (Figure 1).

### 4.3. Lipid Extraction and ESI-MS/MS Analysis

The process of lipid extraction, ESI-MS/MS analysis, and quantification was performed as described previously [17,19]. Briefly, the leaves of two or three different plants were cut at each sampling time and immediately transferred to 3 mL of isopropanol with 0.01% butylated hydroxytoluene (BHT) at 75 °C to inhibit lipolytic activity. After 15 min, 1.5 mL of chloroform and 0.6 mL of water were added. The tubes were shaken for 1 h, followed by the removal of the extract. The samples were re-extracted with chloroform/methanol (2:1) with 0.01% BHT three times with 2 h of agitation each time. The remaining plant tissue was heated overnight at 105 °C and weighed. The weights of these extracted and dried tissues are described as the “dry weight” of the plants. Lipid samples were analyzed on a triple quadrupole MS/MS equipped for ESI. Data processing was performed as previously described [17]. The lipids in each class were quantified in comparison to two internal standards of that class, using a correction curve determined between standards. The internal standards include the following: di 14:0-PG, di 20:0-PG, di 12:0-PE, di 23:0-PE, di 12:0-PC, di 24:1-PC, di 14:0-PA, di 20:0-PA, di 14:0-PS, di 20:0-PS, 16:0–18:0-PI, di 18:0-PI, 34:0-MGDG, 36:0-MGDG, 34:0 DGDG, 36:0 DGDG, 14:0 LysoPG, 18:0 LysoPG, 14:0 LysoPE, 18:0 LysoPE, 13:0 LysoPC, and 19:0 LysoPC. Five replicates of each treatment for each genotype were analyzed.

### 4.4. Data Analysis

The Dixon’s Q-test was performed on all data sets and any results that were remote from the other data were removed. The data were subjected to one-way ANOVA analysis (LSD) of variance with SPSS 16.0 (*P* < 0.05). Paired values were subjected to the two-tailed Student’s *t*-test (SPSS version 16.0) to determine statistical significance (*P* < 0.05).

## 5. Conclusions

Our research indicated that although heat acclimation led to a slight decrease of glycerolipids, it indeed reduced the degradation of glycerolipids under heat shock. The alteration of the content of individual molecular species of glycerolipids under moderate heat stress indicated that the synthesis of glycerolipids through the prokaryotic pathway was severely suppressed, whereas that through the eukaryotic pathway was slightly enhanced. In addition, our research also indicated that HSP101 has a minor effect on the alterations in glycerolipids under both types of heat stresses.

## Supplementary Materials

The following are available online at https://www.mdpi.com/2223-7747/9/6/693/s1.

## Abbreviations

DBI	double bond index
DGDG	digalactosyl diacylglycerol
DHS	direct heat shock
HA	heat acclimation
LysoPC	lysophosphatidylcholine
LysoPE	lysophosphatidylethanolamine
LysoPG	lysophosphatidylglycerol
MGDG	Monogalactosyl diacylglycerol
MHS	moderate heat stress
NDHS	non-direct heat shock
PA	phosphatidic acid
PC	phosphatidylcholine
PE	phosphatidylethanolamine
PG	phosphatidylglycerol
PI	phosphatidylinositol
PS	phosphatidylserine

## References

[1] Christensen J.H., Christensen O.B. (2007). A summary of the PRUDENCE model projections of changes in European climate by the end of this century. Clim. Chang..

[2] Wahid A., Gelani S., Ashraf M., Foolad M.R. (2007). Heat tolerance in plants: An overview. Environ. Exp. Bot..

[3] Zinn K.E., Tunc-Ozdemir M., Harper J.F. (2010). Temperature stress and plant sexual reproduction: Uncovering the weakest links. J. Exp. Bot..

[4] Grover A., Mittal D., Negi M., Lavania D. (2013). Generating high temperature tolerant transgenic plants: Achievements and challenges. Plant Sci..

[5] Jha U.C., Bohra A., Singh N.P. (2014). Heat stress in crop plants: Its nature, impacts and integrated breeding strategies to improve heat tolerance. Plant Breed..

[6] Higashi Y., Saito K. (2019). Lipidomic studies of membrane glycerolipids in plant leaves under heat stress. Pro. Lipid Res..

[7] Tang T., Liu P.L., Zheng G.W., Li W.Q. (2016). Two phases of response to long-term moderate heat: Variation in thermotolerance between Arabidopsis thaliana and its relative Arabis paniculata. Phytochemistry.

[8] Root T.L., Price J.T., Hall K.R., Schneider S.H., Rosenzweig C., Pounds J.A. (2003). Fingerprints of global warming on wild animals and plants. Nature.

[9] Bita C., Gerats T. (2013). Plant tolerance to high temperature in a changing environment: Scientific fundamentals and production of heat stress tolerant crops. Front. Plant Sci..

[10] Welch J.R., Vincent J.R., Auffhammer M., Moya P.F., Dobermann A., Dawe D. (2010). Rice yields in tropical/subtropical Asia exhibit large but opposing sensitivities to minimum and maximum temperatures. Proc. Natl. Acad. Sci. USA.

[11] You L.Z., Rosegrant M.W., Wood S., Sun D.S. (2009). Impact of growing season temperature on wheat productivity in China. Agr. For. Meteorol..

[12] Higashi Y., Okazaki Y., Takano K., Myouga F., Shinozaki K., Knoch E., Fukushima A., Saito K. (2018). HEAT INDUCIBLE LIPASE1 remodels chloroplastic monogalactosyldiacylglycerol by liberating alpha-Linolenic acid in Arabidopsis leaves under heat stress. Plant Cell.

[13] Li Q., Zheng Q., Shen W.Y., Cram D., Fowler D.B., Wei Y.D., Zou J.T. (2015). Understanding the biochemical basis of temperature-induced lipid pathway adjustments in plants. Plant Cell.

[14] Higashi Y., Okazaki Y., Myouga F., Shinozaki K., Saito K. (2015). Landscape of the lipidome and transcriptome under heat stress in Arabidopsis thaliana. Sci. Rep..

[15] Yu B.Z., Li W.Q. (2014). Comparative profiling of membrane lipids during water stress in Thellungiella salsuginea and its relative Arabidopsis thaliana. Phytochemistry.

[16] Chen J.P., Burke J.J., Xin Z.G., Xu C.C., Velten J. (2006). Characterization of the Arabidopsis thermosensitive mutant atts02 reveals an important role for galactolipids in thermotolerance. Plant Cell Environ..

[17] Welti R., Li W.Q., Li M.Y., Sang Y.M., Biesiada H., Zhou H.E., Rajashekar C.B., Williams T.D., Wang X.M. (2002). Profiling membrane lipids in plant stress responses—Role of phospholipase D alpha in freezing-induced lipid changes in Arabidopsis. J. Biol. Chem..

[18] Vigh L., Maresca B., Harwood J.L. (1998). Does the membrane’s physical state control the expression of heat shock and other genes?. Trends Biochem. Sci..

[19] Li W., Wang R., Li M., Li L., Wang C., Welti R., Wang X. (2008). Differential degradation of extraplastidic and plastidic lipids during freezing and post-freezing recovery in Arabidopsis thaliana. J. Biol. Chem..

[20] Dormann P., Benning C. (2002). Galactolipids rule in seed plants. Trends Plant Sci..

[21] Thorlby G., Fourrier N., Warren G. (2004). The sensitive to freezing2 gene, required for freezing tolerance in Arabidopsis thaliana, encodes a beta-Glucosidase. Plant Cell.

[22] Fourrier N., Bedard J., Lopez-Juez E., Barbrook A., Bowyer J., Jarvis P., Warren G., Thorlby G. (2008). A role for SENSITIVE TO FREEZING2 in protecting chloroplasts against freeze-induced damage in Arabidopsis. Plant J..

[23] Moellering E.R., Muthan B., Benning C. (2010). Freezing Tolerance in plants requires lipid remodeling at the outer chloroplast membrane. Science.

[24] Falcone D., Ogas J., Somerville C. (2004). Regulation of membrane fatty acid composition by temperature in mutants of Arabidopsis with alterations in membrane lipid composition. BMC Plant Biol..

[25] Burgos A., Szymanski J., Seiwert B., Degenkolbe T., Hannah M.A., Giavalisco P., Willmitzer L. (2011). Analysis of short-term changes in the Arabidopsis thaliana glycerolipidome in response to temperature and light. Plant J..

[26] Parsell D.A., Kowal A.S., Singer M.A., Lindquist S. (1994). Protein disaggregation mediated by heat-shock protein Hsp104. Nature.

[27] Torok Z., Goloubinoff P., Horvath I., Tsvetkova N.M., Glatz A., Balogh G., Varvasovszki V., Los D.A., Vierling E., Crowe J.H. (2001). Synechocystis HSP17 is an amphitropic protein that stabilizes heat-stressed membranes and binds denatured proteins for subsequent chaperone-mediated refolding. Proc. Natl. Acad. Sci. USA.

[28] Agarwal M., Katiyar-Agarwal S., Grover A. (2002). Plant Hsp100 proteins: Structure, function and regulation. Plant Sci..

[29] Sun W.N., Van Montagu M., Verbruggen N. (2002). Small heat shock proteins and stress tolerance in plants. Biochim. Biophys. Acta-Gene Struct. Expr..

[30] Wu H., Xiao H., Li B. (2003). Research development for plant heat-shock proteins. Biotechnol. Bull..

[31] Wang W.X., Vinocur B., Shoseyov O., Altman A. (2004). Role of plant heat-shock proteins and molecular chaperones in the abiotic stress response. Trends Plant Sci..

[32] Hong S.W., Vierling E. (2000). Mutants of Arabidopsis thaliana defective in the acquisition of tolerance to high temperature stress. Proc. Natl. Acad. Sci. USA.

[33] Queitsch C., Hong S.W., Vierling E., Lindquist S. (2000). Heat shock protein 101 plays a crucial role in thermotolerance in arabidopsis. Plant Cell.

[34] Zhang L., Zhang Q., Gao Y., Pan H., Shi S., Wang Y. (2014). Overexpression of heat shock protein gene PfHSP21.4 in Arabidopsis thaliana enhances heat tolerance. Acta Physiol. Plant..

[35] Mueller S.P., Krause D.M., Mueller M.J., Fekete A. (2015). Accumulation of extra-chloroplastic triacylglycerols in Arabidopsis seedlings during heat acclimation. J. Exp. Bot..

[36] Zhang J.-X., Wang C., Yang C.-Y., Wang J.-Y., Chen L., Bao X.-M., Zhao Y.-X., Zhang H., Liu J. (2010). The role of arabidopsis AtFes1A in cytosolic Hsp70 stability and abiotic stress tolerance. Plant J..

[37] Pearcy R.W. (1978). Effect of growth temperature on fatty acid composition of leaf lipids in a *Atriplex lentiformis* (Torr.) Wats. Plant Physiol..

[38] Zaharieva I., Markova T., Velitchkova M. (1998). Thylakoid Membrane Fluidity Changes the Response of Isolated Pea Chloroplasts to High Temperature.

[39] Wang X.M., Li W.Q., Li M.Y., Welti R. (2006). Profiling lipid changes in plant response to low temperatures. Physiol. Plant..

[40] Munnik T., Irvine R.F., Musgrave A. (1998). Phospholipid signalling in plants. Biochim. Biophys. Acta-Lipids Lipid Metab..

[41] Laxalt A.M., Munnik T. (2002). Phospholipid signalling in plant defence. Curr. Opin. Plant Biol..

[42] Wang X.M. (2004). Lipid signaling. Curr. Opin. Plant Biol..

[43] Ryu S.B. (2004). Phospholipid-derived signaling mediated by phospholipase A in plants. Trends Plant Sci..

[44] Millar A.A., Smith M.A., Kunst L. (2000). All fatty acids are not equal: Discrimination in plant membrane lipids. Trends Plant Sci..

[45] Li Y., Zheng G.W., Jia Y.X., Yu X.M., Zhang X.D., Yu B.Z., Wang D.D., Zheng Y.L., Tian X.J., Li W.Q. (2014). Acyl chain length of phosphatidylserine is correlated with plant lifespan. PLoS ONE.

[46] Fadok V.A., Voelker D.R., Campbell P.A., Cohen J.J., Bratton D.L., Henson P.M. (1992). Exposure of phosphatidylserine on the surface of apoptotic lymphocytes triggers specific recognition and removal by macrophages. J. Immunol..

[47] Gasulla F., vom Dorp K., Dombrink I., Zahringer U., Gisch N., Dormann P., Bartels D. (2013). The role of lipid metabolism in the acquisition of desiccation tolerance in Craterostigma plantagineum: A comparative approach. Plant J..

[48] Deme B., Cataye C., Block M.A., Marechal E., Jouhet J. (2014). Contribution of galactoglycerolipids to the 3-dimensional architecture of thylakoids. FASEB J..

[49] Tsvetkova N.M., Horvath I., Torok Z., Wolkers W.F., Balogi Z., Shigapova N., Crowe L.M., Tablin F., Vierling E., Crowe J.H. (2002). Small heat-shock proteins regulate membrane lipid polymorphism. Proc. Natl. Acad. Sci. USA.

[50] Browse J., Warwick N., Somerville C.R., Slack C.R. (1986). Fluxes through the prokaryotic and eukaryotic pathways of lipid-synthesis in the 16:3 plant Arabidopsis thaliana. Biochem. J..

[51] Heemskerk J.W.M., Storz T., Schmidt R.R., Heinz E. (1990). Biosynthesis of digalactosyldiacylglycerol in plastids from 16-3 and 18-3 Plants. Plant Physiol..

[52] Balfagon D., Sengupta S., Gomez-Cadenas A., Fritschi F.B., Azad R.K., Mittler R., Zandalinas S.I. (2019). Jasmonic acid is required for plant acclimation to a combination of high light and heat stress. Plant Physiol..

[53] Sharma M., Laxmi A. (2016). Jasmonates: Emerging layers in controlling temperature stress tolerance. Front. Plant Sci..

[54] Kazan K. (2015). Diverse roles of jasmonates and ethylene in abiotic stress tolerance. Trends Plant Sci..

[55] Buseman C.M., Tamura P., Sparks A.A., Baughman E.J., Maatta S., Zhao J., Roth M.R., Esch S.W., Shah J., Williams T.D. (2006). Wounding stimulates the accumulation of glycerolipids containing oxophytodienoic acid and dinor-oxophytodienoic acid in Arabidopsis leaves. Plant Physiol..

