# Supplementary material for: Quantitative Profiling of Arabidopsis Polar Glycerolipids under Two Types of Heat Stress
Source: Plants (Basel). 2020 May 29;9(6):693. doi: 10.3390/plants9060693 (PMC7356150; doi:10.3390/plants9060693)
Supplement: Supplementary file 1 [file plants-09-00693-s001.pdf]

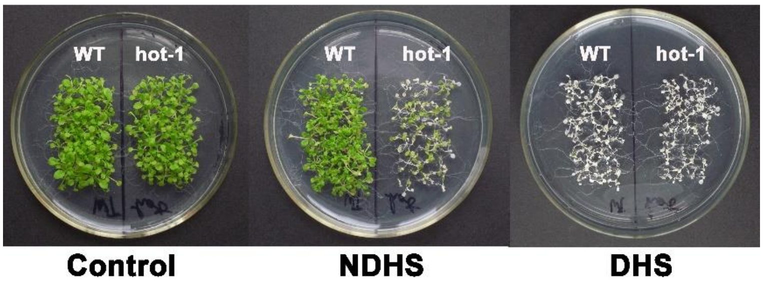

Figure S1. The phenotype of wild-type Arabidopsis and *hot-1* after heat shock. Control: 10-days-old wild-type Arabidopsis and *hot-1* grew under 22°C; NDHS: 10-days-old wild-type Arabidopsis and *hot-1* were exposed to 38°C for 2 hours, returned to 22°C for 1 hour, and 45°C for 3 hours; DHS: 10-days-old wild-type Arabidopsis and *hot-1* were exposed to 45°C for 3 hours. The photographs were taken after recovery at 22°C days for 4 days.

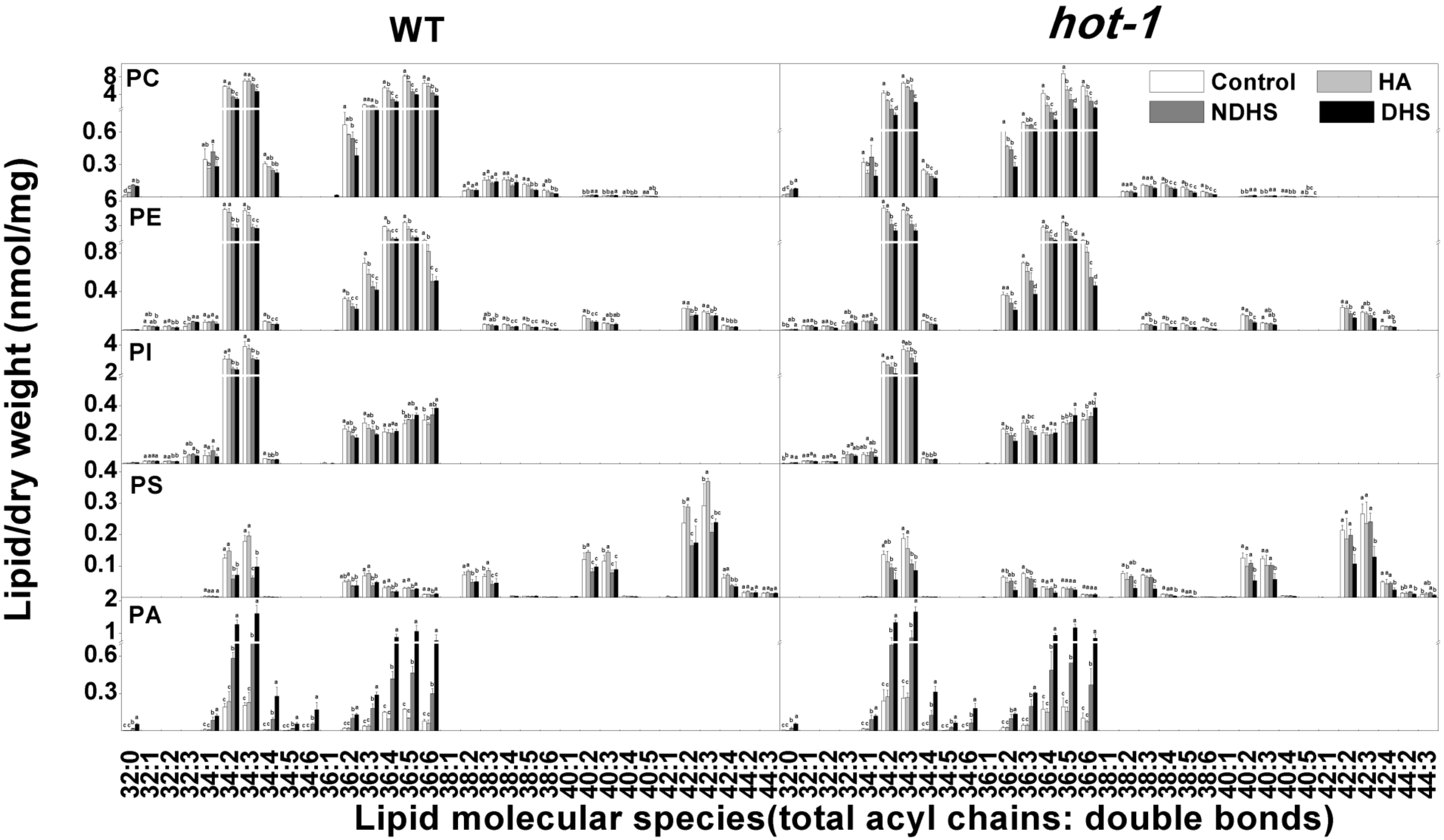

Figure S2. Effects of heat shock on extraplastidic lipid molecular species in wild-type Arabidopsis and *hot-1*. Values shown are the mean ± SE; n = 4 or 5. HA, heat acclimation; NDHS, heat shock with heat acclimation; DHS, direct heat shock. Means with different letters are significantly different according to the least significant difference (LSD) test at $P < 0.05$ under heat shock.

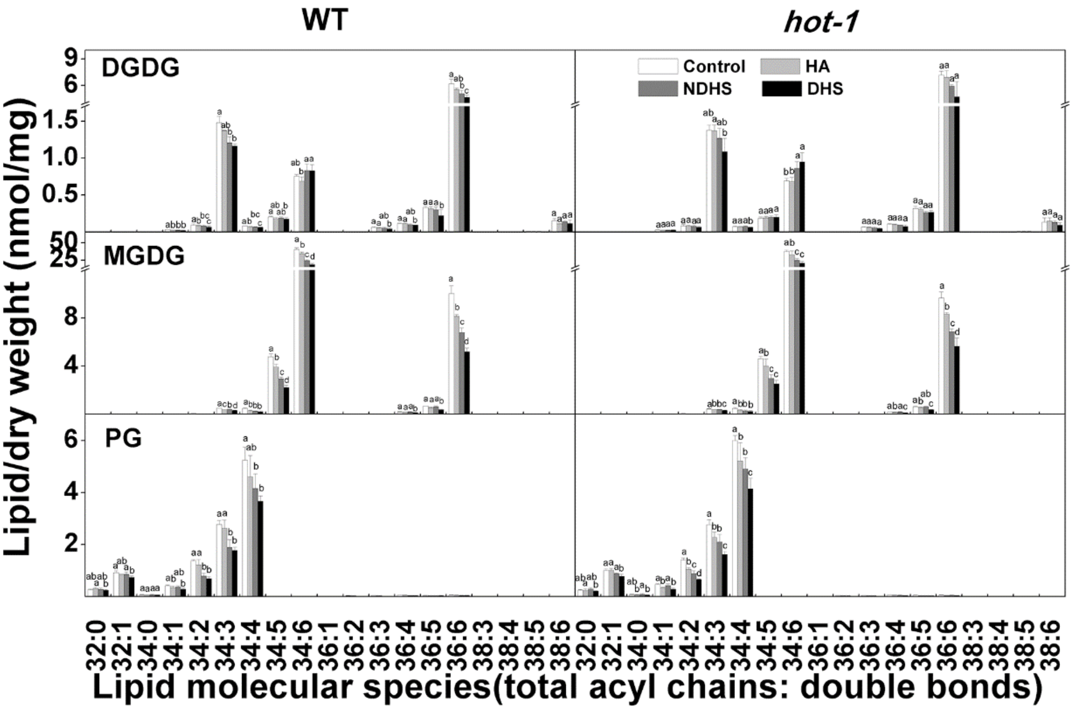

Figure S3. Effects of heat shock on plastidic lipid molecular species in wild-type Arabidopsis and hot-1. Values shown are the mean ± SE; n = 4 or 5. HA, heat acclimation; NDHS, heat shock with heat acclimation; DHS, direct heat shock. Means with different letters are significantly different according to the least significant difference (LSD) test at $P < 0.05$ under heat shock.

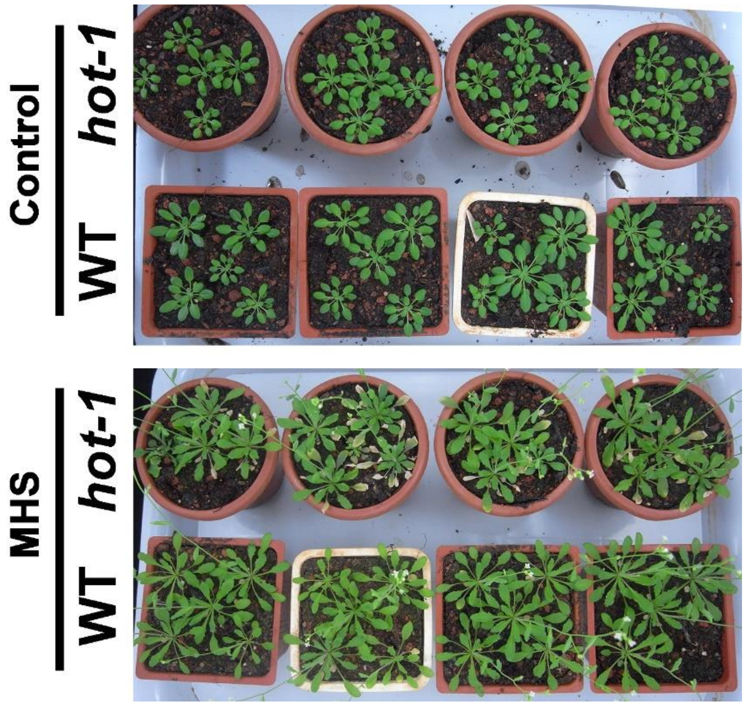

Figure S4. Phenotypes and photosynthetic parameters of wild-type Arabidopsis and *hot-1* under ambient temperature moderate heat stress.
